# Impact of musculoskeletal disability limitations on the economic unmet dental needs in South Korea

**DOI:** 10.1186/s12903-024-04563-5

**Published:** 2024-07-14

**Authors:** Na-Yeon Tak, Jae-In Ryu

**Affiliations:** 1 Department of Preventive and Social Dentistry, Graduate School, Kyung Hee University, Seoul, Republic of Korea; 2 Department of Preventive and Social Dentistry, Kyung Hee University College of Dentistry, Seoul, Republic of Korea

**Keywords:** Musculoskeletal diseases, Oral health, Dental health services, Healthcare disparities

## Abstract

**Background:**

Musculoskeletal disability (MSD) has been identified as having a negative impact on oral health. Patients with MSD have a greater burden of medical expenses and are expected to have an Economic unmet dental need (UDN). This study aimed to conduct a multifactorial analysis based on the Andersen model to determine the extent to which MSD contributes to inequitable dental care use.

**Methods:**

This study used data from the Korea National Health and Nutrition Survey VIII. The study population was 17,903 adults aged 19 years and older. All data were analyzed using IBM SPSS Statistics for Windows version 26 and the level of statistical significance was set at 0.05.

**Results:**

The people with MSD activity limitations were rare as only 3% in this study population. There were significant differences in sex and education as predisposing factors, income, and marital status as enabling factors, and current smoking, daily brushing, and MSD activity limitation as need factors for experiencing economic UDN. MSD activity limitation was associated with 1.5-fold increased odds of Economic UDN with a fully adjusted Anderson’s Behavior Model.

**Conclusions:**

This finding suggests poorer access to dental care among adults with MSDs owing to financial difficulties. It is necessary to explore various ways to address oral health inequalities among adults with MSD activity limitations.

## Background

Oral health is closely related to systemic health, thereby affecting quality of life [1]. Thus, good oral health must be maintained to lead a healthier life [2]. Regular oral examinations and timely access to dental care are essential [3]; otherwise, preventable and simple oral health conditions may become complex and challenging [4, 5]. Oral health is a fundamental right and should be protected as part of comprehensive health [6]; moreover, ensuring access to dental care is also crucial [7]. However, in most countries, dental services are covered less often than other types of health services and are expensive [8, 9]. Unmet dental needs (UDN) are a global public health problem, and financial barriers are one of its major causes [4, 10]. This inability due to financial barriers is a strong indicator of oral health inequality. To reduce oral health inequalities, factors that influence Economic UDN must be identified [10]. People with disabilities are a well-known vulnerable group. Disability refers to a condition that limits a person’s ability to conduct activities of daily living, participate in activities, or interact with others [11]. Oral health is the most commonly unmet health need of people with disabilities [12–14]. Moreover, these people tend to have poorer oral health than people without disabilities [15].

Musculoskeletal disability (MSD) causes problems with bones, muscles, joints, and connective tissue and significantly limits physical activity [16–18]. It is one of the most common and expensive diseases [16, 19], affecting an estimated 1.71 billion people worldwide [20]. People with MSD require social protection and care [4, 21]. MSD is frequent in younger people as well, which is even more problematic in terms of work activity and productivity [22, 23]. Indeed, chronic pain and functional impairment from MSD often lead to loss of productive capacity and unemployment, for example, back pain is reported to be the leading cause of early retirement from work [24]. The Global Burden of Disease, which provides a comprehensive annual assessment of health losses, consistently ranks MSD as the top cause of functional disabilities [23, 25, 26]. It is the most burdensome condition with the longest duration of negative health effects across a lifespan.

Both MSD and oral diseases are potentially serious problems that affect people across their lifespan, but their importance is underestimated because they are often chronic and not life-threatening [27, 28]. MSD has been identified as having a bad influence on oral health. For example, adults with osteoarthritis and rheumatoid arthritis (RA) have difficulties performing the fine motions of toothbrushing, leading to the accumulation of plaque and calculus, which can increase the risk of periodontal infection and dental caries [29–31]. RA patients also tend to have dry mouth which leads to problems in mastication, root caries, dental caries, and periodontal disease [28, 29, 32]. In addition, patients with osteoporosis and cervical spine disorders, are more likely to experience temporomandibular joint dysfunction, decreased alveolar bone density, and mastication disorders due to osteoarticular inflammatory responses [33, 34]. Adults with MSD have higher rates of tooth extraction and filling needs, denture restoration, and prosthodontic needs as well. However, they are less likely to visit the dentist for regular check-ups and preventive care even though they are more likely to be socioeconomically disadvantaged with high dental needs [35, 36].

The Andersen model, a representative health behavior model of disease phenomena, suggests that an individual’s disease behavior, such as UDN, can be explained by a combination of predisposing factors, enabling factors, and need factors [37]. Predisposing factors are the inherent characteristics of an individual that precede the onset of disease behavior. Enabling factors are various environmental factors that make it possible. Needs factors most directly contribute to the development of disease behavior, such as MSD [38]. Although each of these factors varies across studies depending on the operationalization and specific population studied, lower education [39], lower income or job security [40], lower insurance coverage [41], and the presence of illnesses and disabilities [39] have generally been associated with higher odds of experiencing UDN. In other words, patients with MSDs who are more likely to be socioeconomically disadvantaged and have a greater burden of medical expenses beyond dental care are expected to have an Economic UDN. It is important to understand the barriers to care faced by patients with MSD, as it is a global problem that affects both social and oral health [42]. This study aimed to conduct a multifactorial analysis based on the Andersen model to determine the extent to which MSD contributes to inequitable dental care use.

## Methods

### Study design and participants

This study used data from the Korea National Health and Nutrition Survey (KNHANES VIII, 2019–2021), which is a nationwide survey under Article 16 of the National Health Promotion Act conducted every three years in a rotating survey method [43]. The sampling frame was layered according to the size of the area (cities, provinces, and districts) and housing type (general housing or apartments). Finally, 576 districts were surveyed over 3 years, with 10,409 households participating in the study. A total of 22,559 participants were recruited, with a response rate of 74.0%. Among them, 17,903 people were adults aged 19 years and older, which was 79.4% of all participants. This study used data from the Korea National Health and Nutrition Survey (KNHANES VIII, 2019–2021). The KNHANES was conducted with participants’ informed consent by themselves or a legal guardian for those aged under 18 after approval by the Research Ethics Review Committee for the Korea Disease Control and Prevention Agency (KDCA) (IRB No. 2018-01-03-C-A, 2018-01-03–2 C-A, 2018-01-03–3 C-A). This analytical study was approved again by the Institutional Review Board of Kyung Hee University (IRB No. KHSIRB-21-337) and given exemption from review because the retrospective analysis included the dataset from a national survey and did not contain personally identifiable information. All methods were performed following the survey guidelines and regulations.

### Variables

**Fig. 1:**
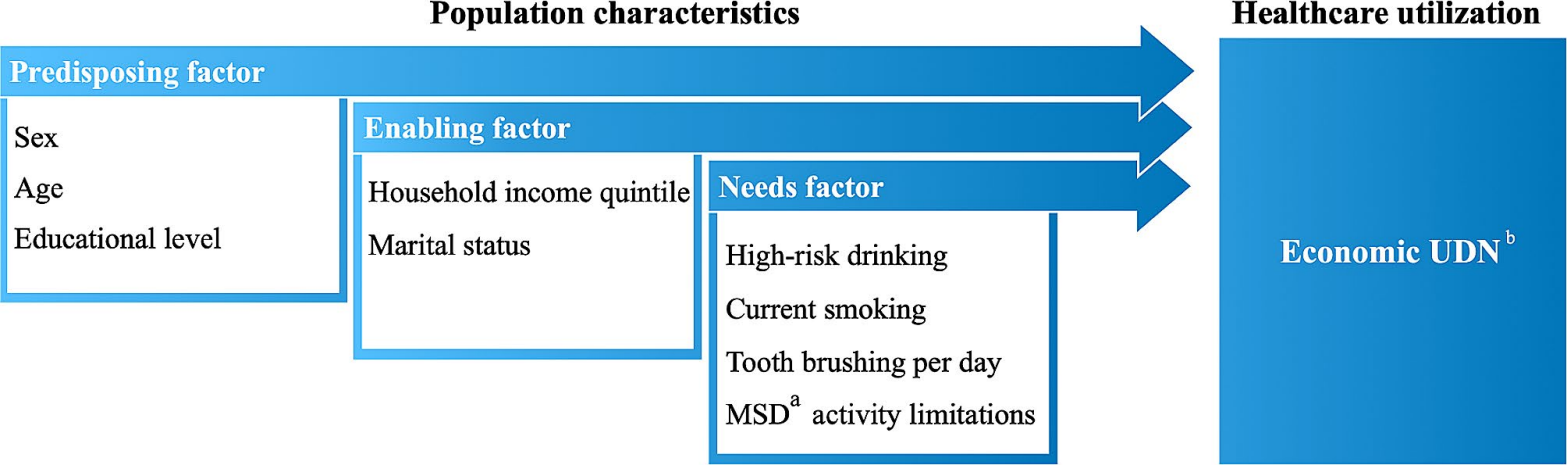
Model of healthcare utilization based on the Andersen conceptual framework a: MSD (Musculoskeletal disability); b: Economic UDN (Unmet Dental Needs due to Economic reason

The analytical model used in this study, which is based on the Andersen model is shown in Fig. 1. The selection of each factor is based on previous studies that analyzed the factors influencing the UDN using the Andersen model [39, 40, 44]. In the Andersen model, the dependent variable was an individual’s health or disease behavior, such as access to health care. In this study, it was an Economic UDN. This was the answer to a two-step question, (1) “Have you ever needed dental care in the past year but did not receive it? If answered ‘yes’, then (2) “Why is the main reason you needed dental care but did not receive it?” as “economic reasons (because the dental costs were unaffordable).” Population characteristics were divided into three categories: predisposing characteristics, enabling resources, and needs. The predisposing factors were sex, age, and education. The enabling factors were income level and marital status. The needs factors were high-risk drinking, current smoking, daily tooth brushing, and restricted MSD activity. Age was divided into three groups: young adults (20 ~ 44), middle-aged (45 ~ 64), and elderly adults (65 + years). Educational level was grouped as a university degree or higher, high school degree, or middle school degree or lower. Personal income level was expressed as quintiles and marital status was labeled as “yes” if there was a spouse and they lived together and “no” if there was a spouse, but they were separated or no longer living together due to widowhood or divorce. Current smoking status was defined according to the KNHANES guidelines as having smoked more than five packs (100 cigarettes) in his/her lifetime. The answer was dichotomized as “yes” if the respondent currently smoked regular cigarettes and “no” if the respondent smoked fewer than five packs of regular cigarettes in his/her lifetime, did not currently smoke, or had never smoked. Daily toothbrushing was classified by two times. MSD experience was defined as “experienced” if the respondent ticked at least one MSD-related illness item and “not experienced” if the respondent ticked none of the items in the health questionnaire, “What are the reasons for the activity limitations in your daily activities?”. MSD-related illnesses were defined as arthritis, rheumatism, back and neck problems, and knee and leg pain, based on previous studies on musculoskeletal disorders [18].

### Analysis

Since the KNHANES is a national sample survey, the analysis reflects a complex sampling design, including weighting. This study applied complex sample analysis by considering complex sampling design factors, such as strata, clusters, and integration weights. A frequency analysis was conducted to determine the general characteristics of all participants, and a cross-tabulation analysis was conducted to determine whether there was a difference in the experience of Economic UDN according to the variables in each categorical factor. Logistic regression analysis was conducted by sequentially introducing variables to determine how the influence changed according to each category of population characteristic. To determine the correlation between the variables in the model, the Variance Inflation Factor (VIF) was analyzed. VIF > 10 indicated the presence of multicollinearity [45]. All data were analyzed using IBM SPSS Statistics for Windows, version 26 (IBM Corp., Armonk, N.Y., USA), and the level of statistical significance was set at 0.05.

## Results

Complex sample frequency and cross-tabulation analyses were conducted to identify differences in Economic UDN experiences based on the participants’ general characteristics (Table 1). There were more female participants (51.4%) than male participants. Young people (41.1%) and middle-aged people (40.5%) were similarly represented, whereas the elderly (18.3%) were the least. The proportion of those with a college degree or higher was almost half (43.5%). The proportion of participants who lived alone was 14%, risky drinkers (12.3%), and current smokers (18.5%). Most of the participants brushed more than twice a day (92.4%). The people with MSD activity limitations were rare (2.9%). The percentage of adults who reported not being able to meet their dental needs due to financial reasons during the past year was 8.5%. The proportion of people with Economic UDN difficulties was higher if the participants were female, older, and had lower educational levels as predisposing factors (*p* < 0.05). Regarding the enabling factors, people with lower income and those living alone had higher rates of Economic UDN (*p* < 0.05). Among the need factors, current smoking and brushing less than twice daily were associated with higher rates of Economic UDN (*p* < 0.05). However, there were no statistically significant differences in high-risk drinking between the two groups (*p* > 0.05). The prevalence of Economic UDN was higher among those with MSD activity limitations (*p* < 0.05).

**Table 1:** Characteristics of the study population by Economic UDN

Variables	Total		Economic UDN^b^			
	*N*	Weighted %	*n*	Weighted %	(SE)	*p*-value
Total	14,046	100	1,261	8.5	(0.3)	
Predisposing factor						
Sex						
Male	6,123	48.6	502	7.9	(0.4)	0.036
Female	7,923	51.4	759	9.0	(0.4)	
Age						
20–44	4,606	41.1	280	6.2	(0.5)	< 0.001
45–64	5,533	40.5	526	9.2	(0.5)	
65 and more	3,907	18.3	455	12.0	(0.6)	
Educational level						
University and more	5,163	43.5	229	4.6	(0.4)	< 0.001
High school	4,520	36.9	439	9.4	(0.5)	
Middle school and below	3,607	19.6	503	14.6	(0.7)	
*Missing*	765					
Enabling factor						
Household income quintile						
5th	2,919	21.2	83	2.3	(0.3)	< 0.001
4th	2,840	20.4	133	4.1	(0.4)	
3rd	2,792	20.0	233	7.6	(0.6)	
2nd	2,725	19.3	333	11.1	(0.7)	
1st	2,717	19.1	478	18.0	(0.9)	
Missing	53					
Marital status						
With family	9,499	86.0	705	7.1	(0.4)	< 0.001
Alone	2,044	14.0	360	18.1	(1.0)	
Missing	2,503					
Needs factor						
High-risk drinking						
No	12,509	87.7	1,112	8.4	(0.4)	0.346
Yes	1,535	12.3	149	9.2	(0.9)	
Missing	2					
Current smoking						
No	11,789	81.5	966	7.6	(0.3)	< 0.001
Yes	2.257	18.5	295	12.3	(0.8)	
Tooth brushing per day						
Twice and more	12,871	92.4	1,082	7.9	(0.3)	< 0.001
Less than twice	1,175	7.6	179	14.8	(1.3)	
MSD^a^ activity limitations						
No	13,493	97.1	1,153	8.1	(0.3)	< 0.001
Yes	553	2.9	108	20.0	(2.1)	

a: MSD (Musculoskeletal disability); b: Economic UDN (Unmet Dental Needs due to Economic reasons

To examine the impact of influencing factors on Economic UDN, hierarchical multiple logistic regression analysis was conducted (Table 2). All values of VIF between the variables were less than 10; therefore, multicollinearity was not observed. First, unadjusted analyses of the predisposing, enabling, and needs variables showed statistical significance in the odds of experiencing Economic UDN, except for high-risk drinking (*p* < 0.05). The odds of experiencing Economic UDN were 2.8 times higher for those with activity limitations due to MSD than for those without (*p* < 0.001). The first model analysis, including predisposing factors and MSD activity restriction, only showed that education level had a significant differential effect on experiencing Economic UDN. High school graduates were 2.1 times more likely to experience Economic UDN than those with a university degree or higher and 3.4 times for those with a middle school degree or less (*p* < 0.001). In Model 2, which included predisposing or enabling factors and MSD activity limitations together, education as a predisposing factor, income and marital status as enabling factors, and MSD activity restriction variables showed statistical significance. The odds of experiencing Economic UDN according to household income showed incremental increase: 1.7, 2.8, 4.5, and 6.4 times higher in each quintile to the baseline income (*p* < 0.01). Those living alone had a 1.8 times higher risk of Economic UDN than those living with a spouse or partner (*p* < 0.001). In Model 3, which included all the factors, there were significant differences in sex and education as predisposing factors, income, and marital status as enabling factors, and current smoking, daily brushing, and MSD activity limitation as need factors. The effect of sex was statistically significant only in Model 3 with 1.2 times higher for women than for men (*p* < 0.05). MSD activity limitation was associated with 2.8-fold increased odds of Economic UDN in the unadjusted model, 2.0 in Model 1, 1.6 in Model 2, and 1.5-fold in Model 3 with a fully adjusted model (*p* < 0.01).

**Table 2:** Complex sample logistic regression analysis regarding influencing factors of economic unmet dental need (UDN)

(= Reference)	Unadjusted			Model1			Model2			Model3		
	OR	95% CI	*p*-value	OR	95% CI	*p*-value	OR	95% CI	*p*-value	OR	95% CI	*p*-value
Predisposing factor												
Sex (= Male)	1.000			1.000			1.000			1.000		
Female	1.144	1.009–1.298	0.036	1.035	0.903–1.186	0.620	1.013	0.857–1.199	0.876	1.226	1.011–1.486	0.038
Age (= 20–44)	1.000			1.000			1.000			1.000		
45–64	1.529	1.259–1.857	< 0.001	1.165	0.950–1.430	0.142	1.240	0.940–1.635	0.127	1.307	0.993–1.722	0.057
65 and more	2.065	1.719–2.480	< 0.001	0.969	0.760–1.236	0.799	1.204	0.878–1.650	0.249	1.335	0.972–1.834	0.074
Educational level (= University+)	1.000			1.000			1.000			1.000		
High school	2.172	1.764–2.675	< 0.001	2.116	1.711–2.617	< 0.001	1.642	1.262–2.137	< 0.001	1.551	1.192–2.019	0.001
Middle school and below	3.593	2.949–4.376	< 0.001	3.388	2.657–4.322	< 0.001	2.040	1.538–2.706	< 0.001	1.865	1.404–2.477	< 0.001
Enabling factor												
Household income quintile (= 5th)	1.000						1.000			1.000		
4th	1.831	1.329–2.523	< 0.001				1.732	1.211–2.478	0.003	1.727	1.206–2.473	0.003
3rd	3.471	2.522–4.777	< 0.001				2.889	2.027–4.119	< 0.001	2.827	1.984–4.026	< 0.001
2nd	5.327	4.015–7.067	< 0.001				4.528	3.270–6.270	< 0.001	4.443	3.206–6.158	< 0.001
1st	9.299	6.878–12.572	< 0.001				6.469	4.608–9.081	< 0.001	6.215	4.424–8.731	< 0.001
Marital status (= with family)	1.000						1.000			1.000		
Alone	2.864	2.414–3.399	< 0.001				1.882	1.547–2.290	< 0.001	1.811	1.494–2.195	< 0.001
Needs factor												
High-risk drinking (= No)	1.000									1.000		
Yes	0.903	0.729–1.117	0.346							0.953	0.715–1.271	0.743
Current smoking (= No)	1.000									1.000		
Yes	1.698	1.437–2.006	< 0.001							1.575	1.262–1.965	< 0.001
Tooth brushing per day (= twice+)	1.000									1.000		
Less than twice	2.009	1.625–2.484	< 0.001							1.469	1.144–1.885	0.003
MSD^a^ activity limitations (= No)	1.000			1.000			1.000			1.000		
Yes	2.829	2.139–3.742	< 0.001	2.009	1.501–2.687	< 0.001	1.554	1.142–2.116	0.005	1.528	1.126–2.073	0.007
Nagelkerke r²				0.048			0.121			0.128		

a: MSD (Musculoskeletal disability); b: Economic UDN (Unmet Dental Needs due to Economic reasons

*Model 1: adjusted for predisposing factors and MSD activity limitation in Needs factors, Model 2: Model 1 plus Enabling factors, Model 3: Model 2 plus needs factors

## Discussion

MSDs mostly have adverse effects on oral health. Patients with MSDs may be at risk of the financial burden of UDN due to reduced productivity and ongoing medical expenses. This study determined the likelihood of Economic UDN among adults with MSDs and examined multiple influencing factors using the Andersen model.

Adults with MSDs were 1.5 times more likely to experience Economic UDN than those without MSD. This finding is similar to that of previous research on dental care utilization among disabled people, indicating poorer access to dental care among adults with MSDs [14]. A study from 15 European Union countries found that MSDs were a major cause of lost working days, 50% of absences over three days, 49% of absences over two weeks, and approximately 60% of early retirements [46]. Work loss and unemployment due to health problems can cause financial difficulties and social isolation. Patients experiencing economic hardship generally tend to forgo dental services [47], particularly vulnerable populations [48]. Dental treatments are mainly not or less covered by governments for health care, so the economic condition has a great impact on the availability of dental services. UDN due to economic reasons can be diminished when the personal economic conditions are improved, or the governments provide financial support. Therefore, governmental support for the dental treatment of vulnerable groups, including those with disabilities, is required.

Among the enabling factors, household income was the most influential factor associated with Economic UDN in the Andersen model. Moreover, a lower income level indicates a higher probability of experiencing Economic UDN by up to 6.21 times. Income has the greatest impact on UDN [49], and the unmet need for dental care is more pronounced in groups with greater economic hardship because of poor public coverage [50]. Out-of-pocket expenses for dental care are higher in South Korea. According to the 2016 World Health Organization (WHO) Health Data, dental coverage in major Organization for Economic Co-operation and Development (OECD) countries was 73.1% on average, while that of Korea was 56.5% [51]. This risk is greater for patients with MSD because they are more likely to have difficulties finding jobs or working life. Dental coverage should be extended to alleviate this burden and increase access to dental care for vulnerable people accordingly.

Men were less likely to experience Economic UDN than women among the predisposing factors. This finding is similar to those of previous studies that explained the reason for the lower incomes for women than those for men [52]. Korea has the largest gender wage gap among the OECD countries [53, 54]. MSDs are more frequent in women; however, the rate of MSD compensation support for female workers was only a quarter of that of male workers [55, 56]. Women with disabilities are more likely to be part-time or temporary employees, putting them at greater risk of living in poverty [57]. These sex inequalities may contribute to the deterioration of inequalities in Economic UDN experienced by people with MSDs.

Those living alone were slightly more likely to experience Economic UDN than those living with a partner or spouse. Married people generally showed higher rates of UDN than single or widowed people [47, 58]. Having someone to live with increases awareness of health care and healthy behaviors, while people living alone tend to lack financial support and help for health care from family members [59, 60]. People with disabilities are mainly cared for by family members; therefore disabled people without family members or cohabitants experience difficulties in daily life, including health care [61]. It is even more devastating for people with MSDs, as they are more likely to experience poverty [16] and difficulties leaving the house owing to physical dysfunction [46]. To ensure dental care for people with MSDs who live alone, policies that support caregivers who can provide appropriate oral health information and accompany them during dental visits are necessary.

Regarding the needs factor, current smokers were more likely to experience Economic UDN than non-smokers, which is similar to the findings of previous studies [62, 63]. Cigarette use is a major factor that negatively affects general and oral health. Smokers are more likely to have poor oral health and higher unmet needs for dental care [64–66]. Furthermore, smoking has serious adverse effects on bones, muscles, tendons, ligaments, and nerves, aggravating MSDs. The American Academy of Orthopedic Surgeons strongly recommends that individuals with MSDs quit smoking. Therefore, future oral health programs for MSDs should consider smoking cessation.

The odds of experiencing Economic UDN were higher for those who brushed less than twice a day than for those who brushed more than twice a day, which is similar to the findings of previous studies [67]. People with MSDs brush less often than the general population [68]. They tend to experience stiffness and pain in their finger joints, which is associated with dysfunction [69, 70]. This makes it difficult for them to maintain their oral hygiene, leading to higher plaque accumulation [68]. Experts recommend the use of high-fluoride toothpaste, chlorhexidine mouthwash, and electric toothbrushes for individuals with limited mobility due to joint pain [71]. Therefore, it is necessary to provide more oral health information to people with MSDs.

The limitations of this study were as follows. First, the KNHANES used in this study is a cross-sectional study; thus, the timing of the observed outcomes cannot be identified. Further research is needed to determine the time order between the diagnosis of MSDs and the occurrence of UDN using longitudinal data, such as panel data. Second, this study focused on Economic UDN and did not explore other causes of UDN. Several UDN reasons, such as lack of time, mildness of symptoms, and inconvenience of transport, have been investigated in the KNHANES; therefore, it would be meaningful to examine various reasons for UDN that are predominantly experienced by people with MSDs. Lastly, this study only used survey data from the KNHANES and did not include diagnostic data. Future studies should combine survey data and MSD diagnosis or dental examination data to better understand the relationship between dental healthcare utilization and actual oral health status among people with MSDs. This study confirmed that MSD activity limitations affect Economic UDN using Korean national-level research data and comprehensively examined and suggested various influencing factors. The findings of this study may provide a basis for institutional policies and intervention programs to address oral health inequalities among patients with MSDs.

## Conclusions

This study analyzed the impact of MSD activity limitations on Economic UDN in adults using the 8th KNHANES. Adults with MSD were more likely to have UDN due to economic reasons even after adjusting all factors in Anderson’s behavior model. This finding suggests poorer access to dental care among adults with MSDs owing to financial difficulties. It is necessary to explore various ways to address oral health inequalities among adults with MSD activity limitations, for example, providing socioeconomic support, dental care services, or oral health promotions.

## Data Availability

The 8th KNHANES data that support the findings of this study are available from the Korea Centre for Disease Control and Prevention Agency (KCDC). Restrictions apply to the availability of these data, which were used under license for this study. Data are available at https://knhanes.kdca.go.kr/knhanes/eng/index.do with the permission of the KCDC.

## Abbreviations

MSD: Musculoskeletal Disability
UDN: Unmet Dental Need
KNHANES: Korea National Health and Nutrition Survey
RA: Rheumatoid Arthritis
KDCA: Korea Disease Control and Prevention Agency
IRB: the Institutional Review Board
SPSS: Statistical Packages for Social Science
WHO: World Health Organization
OECD: Organization for Economic Co-operation, and Development
